# Supplementary material for: Attitudes of women towards intimate partner violence in Guyana: A cross-sectional analytical study
Source: PLoS One. 2024 May 30;19(5):e0303902. doi: 10.1371/journal.pone.0303902 (PMC11139257; doi:10.1371/journal.pone.0303902)
Supplement: S1 File — (PDF) [file pone.0303902.s001.pdf]

S1 Fig: Prevalence of women's attitudes towards intimate partner violence against women in Guyana (2019), by geographic regions:

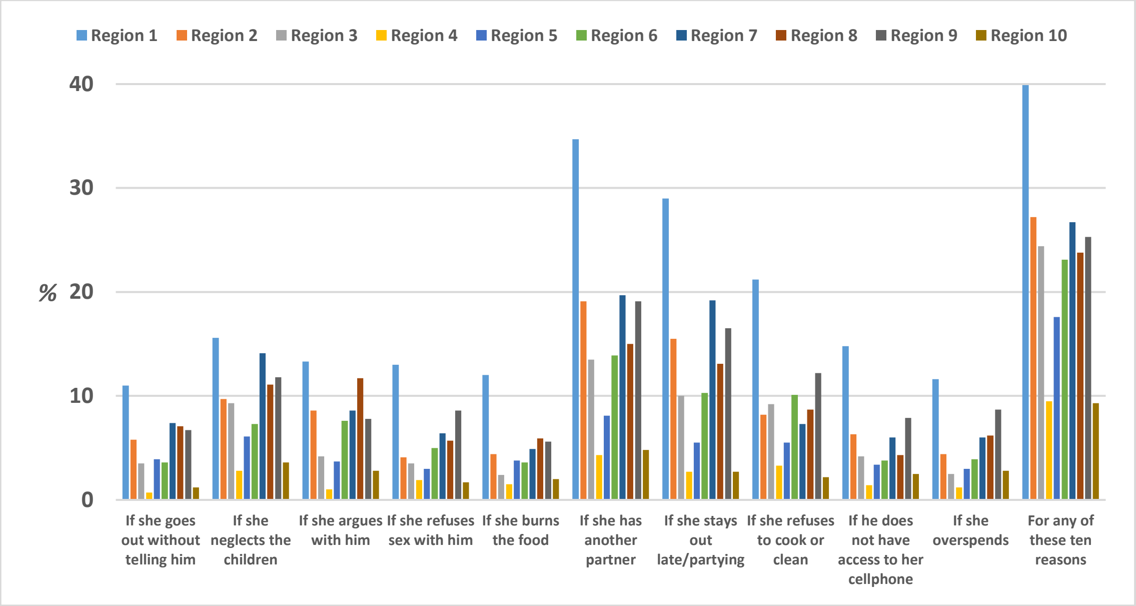

S1 Table: Characteristics of population studied and percentage distribution of the outcomes by selected characteristics.

Independent variables	Description N(%)	If she goes out without telling him	If she neglects the children	If she argues with him	If she refuses sex with him	If she burns the food	If she has another partner	If she stays out late/partying	If she refuses to cook or clean	If he does not have access to her cellphone	If she over spends	For any of the ten reasons
**Area**		***p<0.001***	***p<0.001***	***p<0.001***	***p<0.001***	***p<0.001***	***p<0.001***	***p<0.001***	***p<0.001***	***p<0.001***	***p<0.001***	***p<0.001***
Urban	24.2 (1,424)	1.2 (34)	2.9 (88)	2.2 (56)	2.1 (49)	1.5 (34)	5.6 (146)	3.7 (98)	2.7 (71)	1.5 (48)	1.3 (33)	10.2 (233)
Rural	75.8 (4,463)	3.2 (200)	7.0 (380)	4.4 (276)	3.7 (217)	3.1 (171)	11.1 (648)	8.4 (514)	7.6 (379)	3.9 (234)	3.2 (206)	19.3 (985)
**Location of living**		***p<0.001***	***p<0.001***	***p<0.001***	***p<0.001***	***p<0.001***	***p<0.001***	***p<0.001***	***p<0.001***	***p<0.001***	***p<0.001***	***p<0.001***
Coastal	93.0 (5,476)	2.3 (122)	5.5 (275)	3.4 (185)	2.8 (143)	2.4 (107)	8.7 (460)	6.3 (316)	5.9 (259)	2.8(148)	2.2 (125)	16.1 (777)
Interior	7.0 (411)	8.2(112)	13.5 (193)	10.0 (147)	9.2 (123)	7.5 (98)	23.8 (334)	20.8 (296)	13.6 (191)	9.4 (134)	8.8 (114)	30.1 (421)
**Region**		***p<0.001***	***p<0.001***	***p<0.001***	***p<0.001***	***p<0.001***	***p<0.001***	***p<0.001***	***p<0.001***	***p<0.001***	***p<0.001***	***p<0.001***
Region 1	2.2 (129)	11 (45)	15.6 (73)	13.3 (57)	13 (54)	12 (45)	34.7 (157)	29 (135)	21.1 (93)	14.8 (63)	11.6 (44)	39.9 (183)
Region 2	5.7 (333)	5.8 (31)	9.7 (54)	8.6 (43)	4.1 (22)	4.4 (21)	19.1 (107)	15.5 (78)	8.2 (43)	6.3 (30)	4.4 (25)	27.2 (146)
Region 3	18.9 (1,111)	3.5 (25)	9.3 (72)	4.2 (35)	3.5 (29)	2.4 (17)	13.5 (113)	10.0 (75)	9.2 (67)	4.2 (32)	2.5 (20)	24.4 (192)
Region 4	43.6 (2,566)	0.7 (8)	2.8 (29)	1.0 (12)	1.9 (17)	1.5 (12)	4.3 (46)	2.7 (27)	3.3 (26)	1.4 (14)	1.2 (10)	9.5 (96)
Region 5	6.3 (372)	3.9 (20)	6.1 (34)	3.7 (19)	3.0 (19)	3.8 (17)	8.1 (48)	5.5 (33)	5.5 (28)	3.4 (21)	3.0 (18)	17.6 (91)
Region 6	13.5 (797)	3.6 (32)	7.3 (66)	7.6 (61)	5.0 (46)	3.6 (30)	13.9 (123)	10.3 (89)	10.1 (83)	3.8 (37)	3.9 (37)	23.1 (203)
Region 7	1.7 (94)	7.4 (21)	14.1 (41)	8.6 (27)	6.4 (16)	4.9 (16)	19.7 (59)	19.2 (53)	7.3 (23)	6.0 (20)	6.0 (17)	26.7 (84)
Region 8	0.6 (33)	7.1 (17)	11.1 (26)	11.7 (28)	5.7 (17)	5.9 (13)	15 (38)	13.1 (38)	8.7 (24)	4.3 (14)	6.2 (18)	23.8 (62)
Region 9	2.6 (156)	6.7 (29)	7.8 (53)	7.8 (35)	8.6 (36)	5.6 (24)	19.1 (80)	16.5 (70)	12.2 (51)	7.9 (37)	8.7 (35)	25.3 (112)
Region 10	5.1 (297)	1.2 (6)	2.8 (20)	2.8 (15)	1.7 (10)	2.0 (10)	4.8 (23)	2.7 (14)	2.2 (12)	2.5 (14)	2.8 (15)	9.3 (49)
**Age of woman (in years)**		***p=0.229***	***p=0.015***	***p=0.124***	***p=0.002***	***p=0.009***	***p=0.018***	***p=0.006***	***p=0.009***	***p=0.040***	***P=0.329***	***p=0.041***

Less than 20	16.9 (997)	3.1 (41)	7.3 (96)	3.8 (50)	3.6 (46)	3.5 (39)	11.9 (156)	9.0 (121)	8.2 (88)	4.3 (63)	3.3 (47)	20.5 (228)
20 to 34	47.3 (2,784)	2.5 (101)	5.3 (198)	3.4 (150)	2.5 (103)	2.0 (78)	8.9 (347)	5.8 (259)	5.3 (186)	2.7 (122)	2.5 (105)	16.6 (548)
35 or more	35.8 (2,106)	2.8 (92)	6.6 (174)	4.6 (132)	4.3 (117)	3.4 (88)	10.8 (291)	8.8 (232)	7.3 (176)	3.7 (97)	2.8 (87)	18.5 (442)
**Ethnicity of household head**		***p<0.001***	***p<0.001***	***p<0.001***	***p<0.001***	***p<0.001***	***p<0.001***	***p<0.001***	***p<0.001***	***p<0.001***	***p<0.001***	***p<0.001***
African/Black	30.2 (1,780)	1.0 (20)	2.9 (55)	2.0 (38)	1.9 (28)	0.7 (16)	4.6(90)	2.7 (53)	2.8 (44)	0.9 (25)	1.0 (25)	9.4 (171)
Amerindian	7.3 (429)	7.6 (95)	13.0 (164)	9.7 (126)	8.9 (113)	7.3 (88)	23.1 (280)	19.2 (252)	12.8 (166)	8.9 (114)	8.6 (108)	30.1 (375)
East Indian	43.7 (2,575)	3.5 (89)	8.2 (191)	5.0 (126)	4.2 (97)	4.0 (78)	13.0 (308)	9.8 (230)	8.9 (188)	4.9 (112)	3.6 (83)	22.4 (488)
Mixed race	18.6 (1,093)	1.6 (30)	3.3 (58)	1.7 (42)	1.2 (28)	1.1 (23)	5.5 (115)	4.1 (77)	4.0 (52)	1.2 (31)	1.1 (23)	11.9 (183)
**Marital status**		***p=0.137***	***p=0.996***	***p=0.127***	***p=0.842***	***p=0.352***	***p=0.171***	***p=0.676***	***p=0.779***	***p=0.461***	***p=0.516***	***p=0.032***
Currently married/in union	67.8 (3,984)	3.0 (171)	6.2 (322)	4.3 (245)	3.5 (187)	3.1 (148)	10.9 (570)	8.0 (430)	6.6 (316)	3.3 (190)	2.7 (158)	19.1 (876)
Formerly married/in union	10.1 (594)	0.8 (13)	6.2 (43)	3.1 (27)	2.1 (25)	1.0 (13)	6.2 (67)	5.4 (57)	6.3 (37)	2.5 (22)	2.2 (21)	14.0 (103)
Never married/in union	22.1 (1,296)	2.7 (49)	5.9 (103)	3.1 (59)	3.3 (54)	2.5 (44)	9.6 (157)	6.8 (125)	6.2 (97)	3.8 (69)	3.3 (59)	15.8 (235)
**Women's education**		***p<0.001***	***p<0.001***	***p<0.001***	***p<0.001***	***p<0.001***	***p<0.001***	***p<0.001***	***p<0.001***	***p<0.001***	***p<0.001***	***p<0.001***
Primary	9.3 (539)	6.4 (52)	12.3 (90)	8.6 (71)	1.5 (60)	9.2 (49)	18.5 (136)	14.9 (113)	12.4 (88)	8.4 (53)	5.6 (46)	29.9 (195)
Secondary	72.4 (4,193)	2.5 (162)	6.5 (356)	4.0 (242)	3.3 (186)	2.4 (142)	11.0 (608)	7.9 (464)	7.1 (337)	3.5 (217)	3.0 (180)	19.4 (933)
Tertiary	18.3 (1,063)	1.3 (12)	1.5 (16)	0.7 (9)	1.0 (10)	0.6 (7)	2.2 (28)	1.6 (17)	1.2 (10)	0.3 (4)	0.4 (6)	5.7 (54)
**Wealth index (quintile)**		***p<0.001***	***p<0.001***	***p<0.001***	***p<0.001***	***p<0.001***	***p<0.001***	***p<0.001***	***p<0.001***	***p<0.001***	***p<0.001***	***p<0.001***
Poorest	16.7 (985)	5.4 (131)	9.7 (234)	8.2 (178)	7.2 (148)	5.2 (114)	16.7 (396)	12.9 (335)	9.7 (226)	6.5 (160)	5.4 (135)	25.9 (553)
Second	19.2 (1,130)	3.8 (43)	8.2 (88)	4.5 (59)	3.3 (40)	3.8 (37)	13.7 (161)	10.8 (124)	8.9 (94)	4.9 (54)	4.1 (44)	21.8 (253)
Middle	21.1 (1,240)	2.5 (30)	6.5 (75)	3.6 (49)	2.6 (34)	1.9 (27)	8.9 (112)	5.9 (70)	5.8 (58)	2.4 (30)	2.2 (34)	16.9 (187)

Fourth	22.4 (1,319)	1.2 (18)	3.4 (41)	2.1 (27)	2.0 (23)	1.9 (13)	5.7 (66)	3.7 (44)	3.5 (37)	2.1 (22)	1.2 (13)	11.6 (122)
Richest	20.6 (1,213)	1.2 (12)	3.4 (30)	1.7 (19)	2.2 (21)	1.4 (14)	5.8 (59)	4.7 (39)	5.1 (35)	1.5 (16)	1.3 (13)	11.6 (103)
**Own a mobile phone**		***p<0.001***	***p<0.001***	***p<0.001***	***p<0.001***	***p<0.001***	***p<0.001***	***p<0.001***	***p<0.001***	***p<0.001***	***p<0.001***	***p<0.001***
Yes	87.9 (5,177)	2.2 (160)	5.4 (332)	3.5 (231)	2.7 (181)	2.6 (145)	9.4 (599)	6.6 (437)	5.8 (337)	3.0 (198)	2.4 (161)	16.7 (927)
No	12.1 (710)	6.1 (74)	11.4 (136)	6.8 (101)	7.0 (85)	4.1 (60)	15.4 (195)	13.5 (175)	11.3 (113)	6. (84)	5.4 (78)	27.3 (291)
**Ever used internet**		p<0.001	p<0.001	p<0.001	p<0.001	p<0.001	p<0.001	p<0.001	p<0.001	p<0.001	p<0.001	p<0.001
Yes	65.5 (3,027)	2.0 (61)	6.2 (184)	3.3 (70)	2.3 (72)	2.1 (65)	9.7 (323)	6.5 (230)	6.2 (173)	2.7 (93)	2.2 (79)	17.9 (519)
No	34.5 (1,597)	5.1 (80)	8.7 (135)	7.0 (144)	6.8 (175)	4.9 (122)	14.8 (397)	12.0 (326)	9.7 (239)	5.6 (161)	4.4 (132)	25.0 (581)
**Ever used a computer or a tablet**		***p<0.001***	***p<0.001***	***p<0.001***	***p<0.001***	***p<0.001***	***p<0.001***	***p<0.001***	***p<0.001***	***p<0.001***	***p<0.001***	***p<0.001***
Yes	59.1 (3,478)	1.2 (52)	4.8 (171)	2.1 (95)	1.8 (72)	1.7 (56)	7.2 (265)	4.7 (180)	4.3 (136)	2.1 (82)	1.9 (73)	13.4 (445)
No	40.9 (2,406)	4.9 (182)	8.1 (297)	6.5 (237)	5.6 (194)	4.3 (149)	14.3 (529)	11.4 (432)	9.7 (314)	5.2 (200)	4.1 (166)	24.5 (773)
**Frequency of listening to the radio**		***p=0.065***	***p=0.562***	***p=0.030***	***p=0.008***	***p=0.001***	***p<0.001***	***p<0.001***	***p<0.001***	***p=0.002***	***p=0.001***	***p<0.001***
Not at all	48.7 (2,867)	3.1 (137)	6.6 (255)	4.4 (193)	4.1 (158)	3.9 (135)	12 (446)	9.5 (380)	8.4 (279)	4.0 (170)	3.5 (149)	21.5 (695)
<1 a week	10.3 (605)	2.1 (23)	5.2 (41)	3.0 (28)	3.5 (29)	1.2 (15)	8.7 (69)	5.7 (48)	3.7 (30)	2.7 (24)	1.0 (16)	14.1 (102)
At least 1 a week	12.7 (745)	3.2 (34)	6.4 (68)	4.1 (51)	2.9 (37)	1.8 (24)	9.7 (107)	6.1 (84)	5.9 (60)	3.7 (45)	3.6 (37)	17.4 (179)
Almost everyday	28.4 (1,669)	2.0 (40)	5.4 (104)	3.1 (60)	2.2 (42)	1.7 (31)	7.5 (152)	5.1 (100)	4.5 (81)	2.3 (43)	1.7 (37)	13.4 (242)
**Frequency of watching TV**		***p<0.001***	***p<0.001***	***p<0.001***	***p<0.001***	***p<0.001***	***p<0.001***	***p<0.001***	***p<0.001***	***p<0.001***	***p<0.001***	***p<0.001***
Not at all	19. 1 (1,125)	3.9 (83)	8.1 (162)	6.3 (131)	5.6 (107)	4.1 (84)	12.0 (260)	9.9 (229)	7.4 (159)	4.5 (103)	3.9 (97)	21.8 (397)
<1 a week	9.1 (532)	3.1 (26)	5.7 (39)	3.1 (30)	4.2 (27)	1.8 (19)	10.4 (61)	5.9 (37)	6.6 (36)	3.4 (25)	2.4 (22)	17.2 (96)
At least 1 a week	17.7 (1,040)	2.5 (43)	7.0 (89)	4.1 (56)	3.8 (50)	2.6 (37)	10.6 (143)	6.9 (102)	5.7 (68)	2.6 (42)	3.1 (42)	18.2 (221)
Almost everyday	54.2 (3,185)	2.3 (82)	5.2 (178)	3.1 (115)	2.3 (82)	2.5 (65)	9.2 (330)	7.0 (244)	6.4 (187)	3.2 (112)	2.3 (78)	16.6 (504)

**Total (95%CI)**	**2.7 (2.3-3.2)**	**6.1 (5.4-6.9)**	**3.9 (3.3-4.5)**	**3.3 (2.8-4.1)**	**2.7 (2.2-3.4)**	**10.1 (9.1-11.1)**	**7.4 (6.6-8.3)**	**6.5 (5.7-7.4)**	**3.3 (2.8-4.0)**	**2.7 (2.3-3.3)**	**17.9 (16.6-19.3)**

S2 Table 2: Factors associated with women's attitudes justifying intimate partner violence against women in Guyana by multiple logistic regression analyses.

	If she goes out without telling him[ǂ]		If she neglects the children[¥]		If she argues with him[€]		If she refuses sex with him[β]		If she burns the food[γ]		If she has another partner[δ]		If she stays out late/partying[α]		If she refuses to cook or clean[θ]		If he does not have access to her cellphone[λ]		If she over spends[ϕ]		For any of the ten reasons[ψ]	
Independent variables	COR	AOR	COR	AOR	COR	AOR	COR	AOR	COR	AOR	COR	AOR	COR	AOR	COR	AOR	COR	AOR	COR	AOR	COR	AOR
**Area**																						
Urban	ref**	ref**	ref**	ref**	ref**		ref*	ref£	ref*	ref[£]	ref**	ref**	ref**	ref**	ref**	Ref**	ref**	ref**	ref**	ref**	ref**	ref**
Rural	2.80	2.14	2.58	2.02	2.03	1.62	1.82	1.17	2.08	1.49	2.14	1.64	2.45	1.77	3.02	2.43	2.58	1.96	2.55	2.18	2.14	1.67
**Region**																						
Region 1	ref**	ref£	ref**	ref[£]	ref**	ref[£]	ref**	ref[£]	ref**	ref[£]	ref**	ref**	ref**	ref**	ref**	ref[£]	ref**	ref[£]	ref**	ref**	ref**	ref**
Region 2	0.51	0.72	0.58	0.66	0.61	0.75	0.29	0.39	0.34	0.32	0.42	0.52	0.41	0.50	0.32	0.37	0.38	0.47	0.35	0.42	0.54	0.69
Region 3	0.29	0.44	0.54	0.70	0.28	0.50	0.24	0.48	0.18	0.35	0.27	0.36	0.24	0.33	0.36	0.45	0.24	0.39	0.19	0.27	0.44	0.67
Region 4	0.06	0.11	0.15	0.23	0.07	0.14	0.12	0.25	0.11	0.21	0.07	0.12	0.06	0.10	0.12	0.18	0.08	0.16	0.09	0.16	0.14	0.24
Region 5	0.31	0.39	0.34	0.38	0.24	0.29	0.20	0.27	0.28	0.38	0.15	0.18	0.12	0.15	0.20	0.22	0.19	0.27	0.23	0.29	0.24	0.37
Region 6	0.29	0.44	0.41	0.53	0.52	0.80	0.34	0.57	0.27	0.37	0.27	0.39	0.25	0.34	0.39	0.51	0.22	0.34	0.30	0.47	0.41	0.59
Region 7	0.68	0.96	0.91	1.12	0.65	0.91	0.48	0.54	0.40	0.56	0.45	0.60	0.56	0.74	0.29	0.36	0.38	0.52	0.50	0.64	0.53	0.73
Region 8	0.61	0.58	0.65	0.52	0.86	0.96	0.41	0.40	0.45	0.51	0.30	0.30	0.33	0.31	0.34	0.32	0.24	0.24	0.48	0.45	0.42	0.46
Region 9	0.57	0.55	0.70	0.62	0.54	0.54	0.62	0.54	0.44	0.59	0.42	0.46	0.44	0.47	0.49	0.54	0.48	0.56	0.72	0.76	0.47	0.59
Region 10	0.10	0.37	0.19	0.45	0.18	0.70	0.11	0.34	0.14	0.42	0.08	0.20	0.06	0.16	0.07	0.21	0.14	0.47	0.20	0.63	0.14	0.34
**Age of woman (in years)**																						
Less than 20	ref[£]		ref[£]		ref**	ref[£]	ref[£]		ref*	ref[£]	ref[£]		ref*	ref[£]	ref*	ref[£]	ref[£]		ref[£]		ref[£]	
20 to 34	0.81	-	0.71	-	0.89	0.63	0.69	-	0.56	0.51	0.72	-	0.62	0.74	0.62	0.60	0.62	-	0.76	-	0.74	-
35 or more	0.92		0.89		1.22	0.73	1.20		0.99	0.64	0.90		0.97	0.94	0.88	0.67	0.85		0.87		0.88	
**Ethnicity of household head**																						
African/Black	ref**	ref[£]	ref**	ref[£]	ref**	ref[£]	ref**	ref[£]	ref**	ref**	ref**	ref[£]	ref**	ref**	ref**	ref**	ref**	ref**	ref**	ref[£]	ref**	ref**
Amerindian	8.58	3.19	5.30	2.58	5.32	1.74	5.18	1.82	10.82	4.53	6.79	3.55	9.35	4.37	5.31	2.77	10.80	4.48	9.76	3.85	4.56	2.33
East Indian	3.63	2.66	3.08	2.30	2.56	1.68	2.30	1.65	5.71	3.81	3.22	2.42	4.04	2.86	3.45	2.26	5.71	4.33	3.82	2.96	2.89	2.12
Mixed race	1.69	1.37	1.16	1.01	0.84	0.60	0.65	0.51	1.46	1.15	1.22	1.00	1.56	1.28	1.44	1.25	1.27	1.02	1.17	1.00	1.33	1.14
**Marital status**																						

Currently married/in union	ref*	ref£	ref£		ref£		ref£		ref*	ref£	ref*	ref£	ref£		ref£		ref£		ref£		ref*	ref£
Formerly married/in union	0.27	0.32	1.00	-	0.72	-	0.59	-	0.31	0.24	0.54	0.57	0.66	-	0.94	-	0.75	-	0.83	-	0.69	0.75
Never married/in union	0.90	1.21	0.94		0.71		0.94		0.82	1.00	0.87	1.02	0.84		0.93		1.14		1.24		0.79	0.91
**Women's education**																						
Primary	ref**	ref£	ref**	ref**	ref**	ref*	ref**	ref*	ref**	ref**	ref**	ref**	ref**	ref*	ref**	ref**	ref**	ref**	ref**	ref*	ref**	ref**
Secondary	0.37	0.63	0.50	0.61	0.44	0.65	0.37	0.57	0.25	0.30	0.54	0.72	0.49	0.73	0.54	0.75	0.39	0.50	0.51	0.64	0.56	0.77
Tertiary	0.19	0.76	0.11	0.18	0.07	0.19	0.10	0.15	0.06	0.11	0.10	0.79	0.10	0.26	0.08	0.17	0.04	0.07	0.07	0.14	0.14	0.28
**Wealth index (quintile)**																						
Poorest	ref**	ref*	ref**	ref**	ref**	ref**	ref**	ref£	ref**	ref**	ref**	ref**	ref**	ref**	ref**	ref£	ref**	ref**	ref**	ref*	ref**	ref**
Second	0.62	0.83	0.80	0.89	0.54	0.65	0.46	0.61	0.73	0.79	0.76	0.86	0.72	0.93	0.88	1.08	0.69	0.72	0.77	0.85	0.78	0.88
Middle	0.42	0.67	0.63	0.81	0.42	0.59	0.34	0.72	0.35	0.41	0.48	0.64	0.41	0.60	0.55	0.83	0.33	0.41	0.39	0.49	0.56	0.75
Fourth	0.21	0.39	0.32	0.45	0.23	0.38	0.26	0.66	0.36	0.50	0.29	0.40	0.24	0.38	0.33	0.54	0.29	0.39	0.21	0.29	0.36	0.51
Richest	0.20	0.44	0.32	0.51	0.20	0.38	0.28	0.73	0.26	0.43	0.30	0.48	0.33	0.62	0.48	0.98	0.21		0.23	0.34	0.36	0.59
**Own a mobile phone**																						
Yes	0.35	0.60	0.45	0.65	0.49	0.89	0.39	0.67	0.61	1.03	0.57	0.87	0.45	0.70	0.48	0.67	0.49	0.83	0.42	0.74	0.54	0.76
No	ref**	ref*	ref**	ref*	ref**	ref£	ref**	ref£	ref*	ref£	ref**	ref£	ref**	ref*	ref**	ref*	ref**	ref£	ref**	ref£	ref**	ref£
**Ever used internet**																						
Yes	0.38	0.89	0.69	1.13	0.45	0.84	0.32	0.50	0.42	0.76	0.62	1.00	0.51	0.91	0.61	1.00	0.45	0.87	0.50	0.92	0.65	1.02
No	ref**	ref£	ref*	ref£	ref**	ref£	ref**	ref*	ref**	ref£	ref**	ref£	ref**	ref£	ref*	ref£	ref**	ref£	ref*	ref£	ref**	ref£
**Ever used a computer or a tablet**																						
Yes	0.24	0.34	0.57	0.99	0.31	0.49	0.30	0.55	0.38	0.73	0.47	0.73	0.38	0.62	0.42	0.60	0.39	0.70	0.45	0.82	0.48	0.71
No	ref**	ref**	ref**	ref£	ref**	ref**	ref**	ref*	ref**	ref£	ref**	ref**	ref**	ref*	ref**	ref*	ref**	ref£	ref**	ref£	ref**	ref**
**Frequency of listening to the radio**																						
Not at all	ref£		ref£		ref£		ref£		ref**	ref*	ref*	ref£	ref**	ref£	ref**	ref*	ref£		ref**	ref£	ref**	ref*
<1 a week	0.66	-	0.77	-	0.67	-	0.85	-	0.30	0.34	0.70	0.76	0.58	0.63	0.43	0.42	0.67	-	0.28	0.32	0.60	0.65
At least 1 a week	1.00		0.97		0.97		0.71		0.46	0.54	0.79	0.90	0.61	0.76	0.68	0.84	0.93		1.03	1.26	0.77	0.91
Almost everyday	0.63		0.80		0.68		0.54		0.43	0.53	0.59	0.78	0.51	0.70	0.52	0.67	0.55		0.46	0.62	0.56	0.73

**Frequency of watching TV**																						
Not at all	ref£	-	ref*	ref£	ref**	ref£	ref*	ref*	ref*	ref£	ref£	-	ref*	ref£	ref£	-	ref£	-	ref£	-	ref*	ref£
<1 a week	0.81		0.69	0.81	0.48	0.65	0.73	1.06	0.43	0.37	0.85		0.57	0.87	0.89		0.75		0.61		0.75	0.96
At least 1 a week	0.64		0.86	1.05	0.63	0.84	0.67	0.92	0.61	0.87	0.87		0.68	0.95	0.76		0.56		0.80		0.80	1.03
Almost everyday	0.58		0.63	0.77	0.48	0.66	0.39	0.56	0.59	0.81	0.74		0.68	1.04	0.86		0.69		0.58		0.72	0.91

*p value <0.05, **p value <0.001, £p value>0.05, [ŧ]The final model was adjusted for area, wealth quintile, own a mobile phone and ever use a computer or tablet. [¥]The final model was adjusted for area, level of education, wealth quintile and own a mobile phone. [€]The final model was adjusted for area, level of education, wealth quintile and ever use a computer or tablet. [β]The final model was adjusted for level of education, ever used internet and computer. [γ]The final model was adjusted for level of education, wealth quintile and frequency of listening radio. [δ]The final model was adjusted for area, region, level of education, wealth quintile, and ever use a computer. [α]The final model was adjusted for area, ethnicity, region, level of education, wealth quintile, own a mobile phone, ever used a computer, and frequency of listening radio. [θ]The final model was adjusted for area, ethnicity, level of education, own a mobile phone, ever used a computer, and frequency of listening radio. [λ]The final model was adjusted for area, ethnicity, level of education, and wealth quintile. [ϕ]The final model was adjusted for area, ethnicity, level of education, own a mobile phone, ever used a computed, and frequency of listening radio. The final model was adjusted for area, ethnicity, region, level of education, wealth quintile, ever used a computer, and frequency of listening radio.
