# Supplementary material for: Attitudes of women towards intimate partner violence in Guyana: A cross-sectional analytical study
Source: PLoS One. 2024 May 30;19(5):e0303902. doi: 10.1371/journal.pone.0303902 (PMC11139257; doi:10.1371/journal.pone.0303902)
Supplement: Attachment — Submitted filename: Review.docx [file pone.0303902.s003.docx]

Thanks for this amazing work. I loved to read this manuscript. It is well written an well addressed all the isses.
